# Trajectories in glycated hemoglobin and body mass index in children and adolescents with diabetes using the common data model

**DOI:** 10.1038/s41598-021-94194-5

**Published:** 2021-07-16

**Authors:** Yun Jeong Lee, Sooyoung Yoo, Soyoung Yi, Seok Kim, Chunggak Lee, Jihoon Cho, Soyeon Ahn, Sunkyu Choi, Hee Hwang, Young Ah Lee, Choong Ho Shin, Hyung-Jin Yoon, Kwangsoo Kim, Eunhye Song, Jin Ho Choi, Han Wook Yoo, Young-Hak Kim, Ji Seon Oh, Eun-Ae Kang, Ga Kyoung Baek, Jae Hyun Kim

**Affiliations:** 1 Department of Pediatrics, Seoul National University Children’s Hospital, Seoul, Korea; 2 Department of Pediatrics, Seoul National University College of Medicine, Seoul, Korea; 3 Office of eHealth Research and Businesses, Seoul National University Bundang Hospital, Seongnam, Gyeonggi-do Korea; 4 Division of Statistics, Medical Research Collaborating Center, Seoul National University Bundang Hospital, Seongnam, Gyeonggi-do Korea; 5 Department of Biostatistics, Korea University College of Medicine, Seoul, Korea; 6 Department of Biomedical Engineering, Seoul National University College of Medicine, Seoul, Korea; 7 Transdisciplinary Department of Medicine and Advanced Technology, Seoul National University Hospital, Seoul, Korea; 8 Department of Data Science Research, Innovative Medical Technology Research Institute, Seoul National University Hospital, Seoul, Korea; 9 Department of Pediatrics, Asan Medical Center, University of Ulsan College of Medicine, Seoul, Korea; 10 Department of Information Medicine, Asan Medical Center, University of Ulsan College of Medicine, Seoul, Korea; 11 Division of Cardiology, Department of Internal Medicine, Asan Medical Center, University of Ulsan College of Medicine, Seoul, Korea; 12 Health Innovation Big Data Center, Asan Institute of Life Sciences, Asan Medical Center, Seoul, Korea; 13 Department of Pediatrics, Seoul National University Bundang Hospital, Seongnam, Gyeonggi-do Korea

**Keywords:** Type 1 diabetes, Type 2 diabetes, Paediatric research

## Abstract

We evaluated trajectories of glycated hemoglobin (HbA1c) levels and body mass index z-scores (BMIz) for 5 years after diagnosis among Korean children and adolescents with type 1 diabetes (T1D) or type 2 diabetes (T2D) using the common data model. From the de-identified database of three hospitals, 889 patients < 15 years of age diagnosed with T1D or T2D (393 boys, 664 T1D patients) were enrolled. Diagnosis was defined as first exposure to antidiabetic drug at each center. Compared with T2D patients, T1D patients had lower BMIz at diagnosis (− 0.4 ± 1.2 vs. 1.5 ± 1.4, *p* < 0.001) and 3 months (− 0.1 ± 1.0 vs. 1.5 ± 1.5, *p* < 0.001), and higher HbA1c levels at diagnosis (10.0 ± 2.6% vs. 9.5 ± 2.7%, *p* < 0.01). After 3 months, HbA1c levels reached a nadir of 7.6% and 6.5% in T1D and T2D patients, respectively, followed by progressive increases; only 10.4% of T1D and 29.7% of T2D patients achieved the recommended HbA1c target (< 7.0%) at 60 months. T1D patients showed consistent increases in BMIz; T2D patients showed no significant change in BMIz during follow-up. Peri-pubertal girls with T1D had higher HbA1c and BMIz values. Achieving optimal glycemic control and preventing obesity should be emphasized in pediatric diabetes care.

## Introduction

The incidence of type 1 (T1D) and type 2 diabetes (T2D) in children and adolescents is increasing in Korea^1–3^, similar to global trends^4,5^. Achievement of optimal glycemic control and growth, along with prevention of obesity, is the main goal of care for pediatric T1D and T2D^6,7^. Although intensive insulin therapy for T1D to optimize glycemic control reduces the incidence of microvascular complications, there is concern over excessive weight gain during treatment^8,9^, which may compromise metabolic control and lead to cardiovascular diseases^10,11^. Obesity is highly prevalent in youth with T2D^12^, and changes in body mass index (BMI) have been associated with metabolic control in patients with T2D^13,14^.

In this regard, it is important to monitor the longitudinal patterns of glycemic control and BMI in children and adolescents with diabetes. Based on recent registry data from western countries, the glycated hemoglobin (HbA1c) level and BMI trajectories of youth with T1D showed heterogeneous patterns according to sex, age, pubertal status, and treatment regimen^15–18^, suggesting the need to identify high-risk groups for metabolic deterioration. To date, no study has investigated the long-term trends of the HbA1c level or BMI in Korean children and adolescents with diabetes.

The common data model (CDM) is a shared data model that enables secondary use of longitudinal electronic health record (EHR) data for large-scale observational studies. The Observational Health Data Sciences and Informatics (OHDSI) community supports a distributed research network that enables multicenter research by standardizing and harmonizing observational health data into standard terminologies and structures based on the Observational Medical Outcomes Partnership (OMOP) CDM^19^. In a distributed research network, results of analyses performed by multiple organizations can be efficiently integrated using standardized analytical tools while keeping de-identified data sources secure within each organization.

In this study, we investigated the longitudinal trajectories of the HbA1c level and BMI z-score (BMIz) for 5 years after diagnosis in children and adolescents with T1D and T2D from Korea. To generate reliable real-world evidence, we analyzed longitudinal EHR data sources converted to OMOP CDM at three tertiary hospitals in Korea participating in the OHDSI community.

## Results

### Clinical characteristics of the participants

Table 1 shows the clinical characteristics of the participants according to the type of diabetes. A total of 889 patients (393 boys) were enrolled: 664 (75.0%) with T1D and 225 (25.0%) with T2D patients. The index date of diabetes diagnosis was defined as any first exposure to insulin or hypoglycemic drug. The T1D patients, compared with the T2D patients, comprised fewer boys (41.7% vs. 51.6%, *p* = 0.013), and were younger at diagnosis (mean age, 9.9 ± 3.7 vs. 12.2 ± 1.1 years, *p* = 0.001). The mean HbA1c level was 10.0 ± 2.6% in T1D patients and 9.5 ± 2.7% in T2D patients (*p* = 0.008). The T1D patients, compared with the T2D patients, had a lower BMIz at diagnosis (− 0.4 ± 1.2 vs. 1.5 ± 1.4, *p* < 0.001) and a lower proportion of patients with overweight and/or obesity (9.7% vs. 62.1%, *p* < 0.001). In T2D patients, the most common treatment at 3 months was metformin monotherapy (133/183, 72.7%), which has weight-neutral effects. The other treatments were insulin in combination with metformin (36/183, 19.7%) and insulin alone (14/183, 7.7%). Drugs used in T2D patients at other time points are described in Supplementary Table S1. A comparison of baseline characteristics between patients followed-up and those lost to follow-up at each time point is shown in Supplementary Table S2.

**Table 1:** Baseline characteristics of the study participants.

Characteristics	Total	Type 1 diabetes	Type 2 diabetes	*P values*
Number of patients (%)	889	664 (74.7)	225 (25.3)	–
**Institute**				
SNUBH, n (%)	98	72 (73.5)	26 (26.5)	0.367
SNUH, n (%)	431	331 (76.8)	100 (23.2)	
AMC, n (%)	360	261 (72.5)	99 (27.5)	
Male, n (%)	393 (44.2)	277 (41.7)	116 (51.6)	0.013
Age at diagnosis, years	10.5 (3.5)	9.9 (3.7)	12.2 (1.1)	< 0.001
**Age group**				
0–4 years, n (%)	100 (11.2)	100 (15.1)	0 (0.0)	< 0.001
5–9 years, n (%)	210 (23.6)	181 (27.3)	29 (12.9)	
10–14 years, n (%)	579 (65.1)	383 (57.7)	196 (87.1)	
Body mass index or weight-for-height z-score^a^	0.1 (1.5)	− 0.4 (1.2)	1.5 (1.4)	< 0.001
Normal/Overweight /Obese^a^, n (%)	553/60/100 (77.6/8.4/14.0)	487/37/15 (90.4/6.9/2.8)	66/23/85 (37.9/13.2/48.9)	< 0.001
Glycated hemoglobin^a^, %	9.9 (2.6)	10.0 (2.6)	9.5 (2.7)	0.008

Data are presented as means (standard deviation) for continuous variables and numbers (%) for categorical variables. *p*-values are for Student’s *t*-test (continuous variables) or the chi-squared test (categorical variables).

^a^Body mass index z-scores at baseline were available for 713 patients, and the glycated hemoglobin level at baseline was available for 831 patients.

### Trajectory of the HbA1c level for 5 years after diagnosis

**Figure 1:**
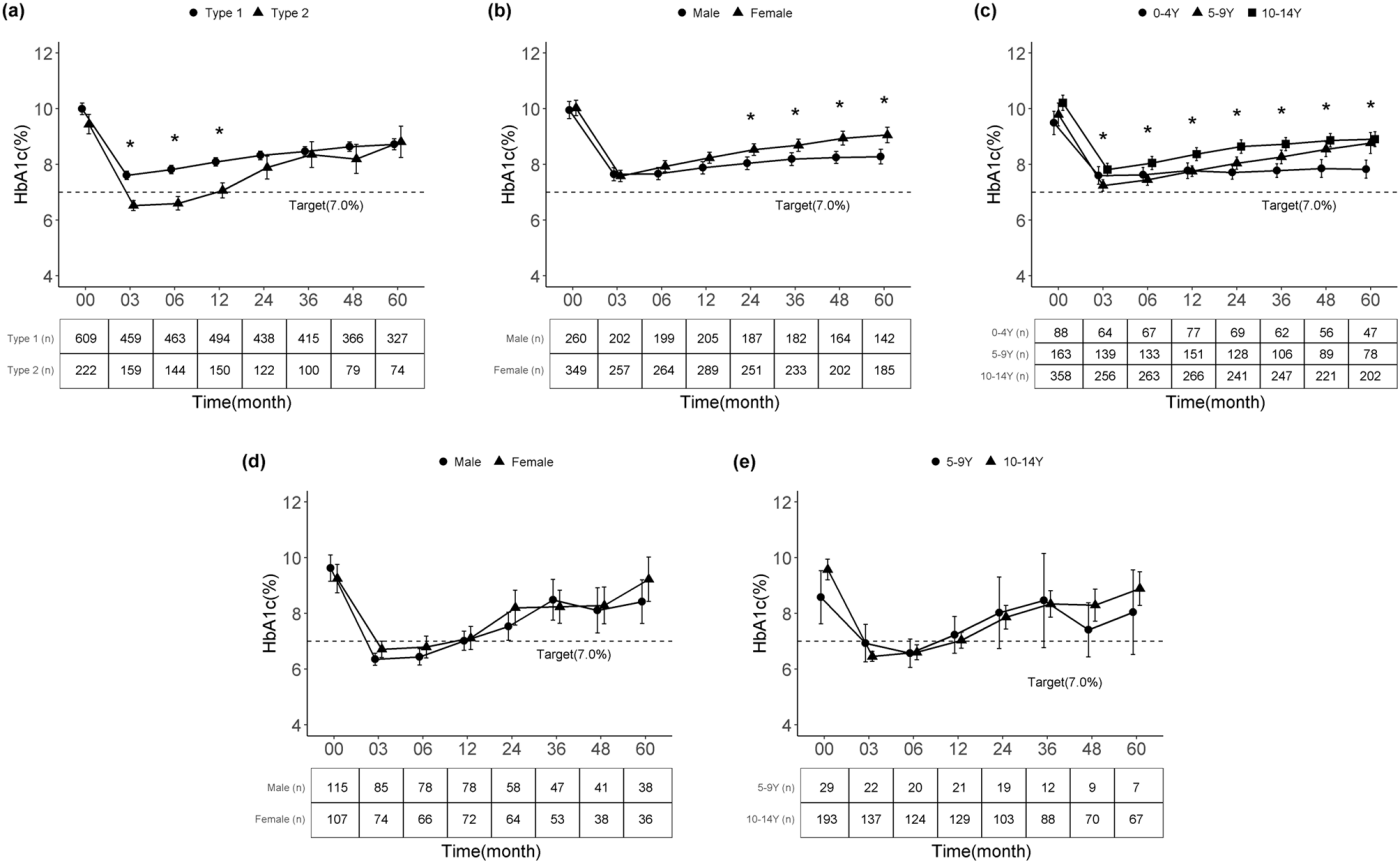
Trajectory of the average glycated hemoglobin level over 5 years after diagnosis according to type of diabetes, sex, and age group. (**a**) All patients according to type of diabetes, (**b**) type 1 diabetes according to sex, (**c**) type 1 diabetes according to age group, (**d**) type 2 diabetes according to sex, and (**e**) type 2 diabetes according to age group. *Bonferroni-corrected *p*-value < 0.05.

The T1D and T2D patients showed a rapid decrease in the HbA1c level during the first 3–6 months, followed by a gradual increase thereafter during the 5-year period after diagnosis (Fig. 1a). The HbA1c level was significantly higher in T1D than T2D patients from diagnosis to 12 months (all *p* < 0.05, Supplementary Table S3).

In T1D patients, the mean HbA1c level decreased from 10.0 ± 2.6% at diagnosis (n = 609) to 7.6 ± 1.7% (n = 459) at 3 months, followed by a progressive increase to 8.7 ± 1.8% (n = 327) at 60 months (Fig. 1a, Supplementary Table S3); only 10.4% of patients attained the HbA1c target of < 7% at 60 months (Supplementary Fig. S1). The HbA1c level was significantly higher at 36 or 60 months than at 3 months (*p* < 0.001 for both, Supplementary Table S4).

Boys and girls showed a significant increase in the HbA1c level from 3 to 60 months (Fig. 1b, Supplementary Table S4), although the HbA1c level was higher in girls than in boys from 24 months after diagnosis (all *p* < 0.05, Fig. 1b). When stratified by age group, the 0–4-year group exhibited little change in the HbA1c level between 3 and 60 months, while the other groups, especially the 5–9-year group, showed gradually increasing trends (Fig. 1c). Comparison of patients diagnosed before and after 2010 showed similar worsening of glycemic control. However, in comparison with patients diagnosed before 2010, those diagnosed after 2010 had lower HbA1c levels until 3 years after diagnosis (data not shown).

In T2D patients, the mean HbA1c level decreased from 9.5 ± 2.7% (n = 222) at diagnosis to a nadir of 6.5 ± 1.2% (n = 159) at 3 months and then began to increase at 6–12 months, reaching 8.8 ± 2.5% (n = 74) at 60 months (Fig. 1a, Supplementary Table S3). The proportion of patients in the good glycemic control group decreased from 75.5% at 3 months to 29.7% at 60 months (Supplementary Fig. S1). The HbA1c level was significantly increased at both 36 and 60 months compared with 3 months (*p* < 0.001 for both, Supplementary Table S4). Boys and girls with T2D showed similar patterns of increasing HbA1c levels from 3 to 60 months (Fig. 1d). No significant difference in the HbA1c trajectory was observed according to age group (Fig. 1e) or year of diagnosis (data not shown).

### Trajectory of BMIz for 5 years after diagnosis

**Figure 2:**
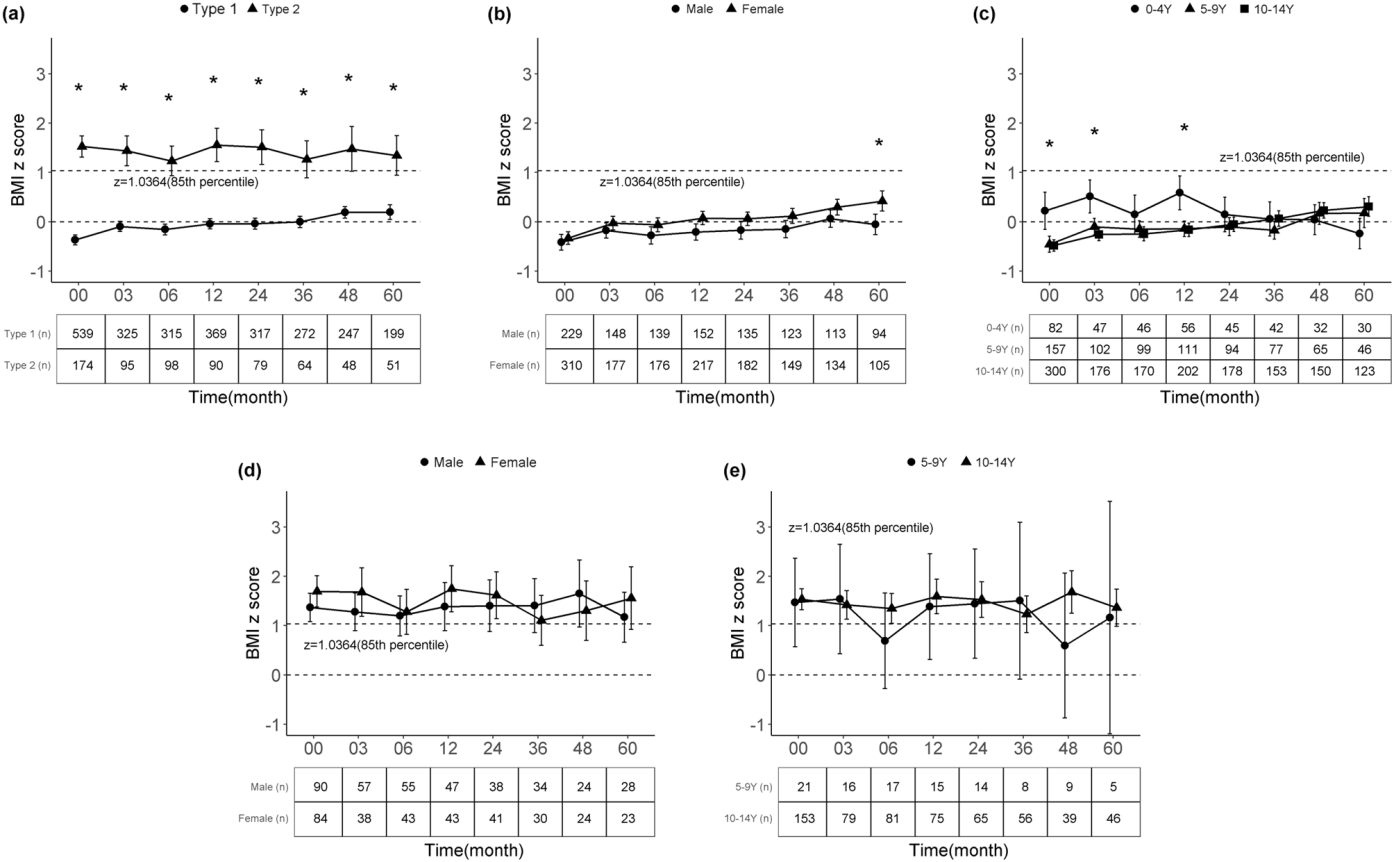
Trajectory of the average body mass index z-score over 5 years after diagnosis according to type of diabetes, sex, and age group. (**a**) All patients according to diabetes type, (**b**) type 1 diabetes according to sex, (**c**) type 1 diabetes according to age group, (**d**) type 2 diabetes according to sex, and (**e**) type 2 diabetes according to age group. *Bonferroni-corrected *p*-value < 0.05.

Figure 2a shows the BMIz trajectory of patients according to type of diabetes over the 5 years from diagnosis. T1D patients exhibited a rapid increase in the BMIz from − 0.4 ± 1.2 (n = 539) at diagnosis to − 0.1 ± 1.0 (n = 325) at 3 months after diagnosis, whereas those with T2D showed an initial decrease in the BMIz from 1.5 ± 1.4 (n = 174) at diagnosis to a nadir of 1.3 ± 1.5 (n = 98) at 6 months. After stabilization, T1D patients showed a gradual increase in the BMIz until 60 months whereas T2D patients showed no significant change in BMIz during follow-up (Fig. 2a). The BMIz was consistently higher in T2D than T1D patients during follow-up (*p* < 0.001 for all time points, Supplementary Table S5). The proportion of patients in the overweight/obesity group increased from 9.5% at 3 months to 21.1% at 60 months among T1D patients, whereas it remained stable (62.1% at 3 months to 58.8% at 60 months) among T2D patients (Supplementary Fig. S2). For T1D, the BMIz was significantly increased at 60 months compared with 3 months (*p* < 0.01, Supplementary Table S6).

In T1D patients, both boys and girls showed a gradual increase in the BMIz with a similar pattern (Fig. 2b); however, the BMIz was higher in girls than boys from diagnosis to 60 months, although the difference was not statistically significant. When stratified by age group, the 0–4-year group showed an initial increase in the BMIz for 12 months, followed by a stable BMIz at 12–60 months; the other groups showed consistently increasing patterns. The 5–9-year group showed a prominent increase in BMIz at 36–60 months (Fig. 2c). No significant difference was seen in the BMIz trajectory according to year of diagnosis (data not shown). For T2D, there were no significant differences in BMIz trajectories during follow-up according to sex (Fig. 2d), age group (Fig. 2e), or years of diagnosis (data not shown).

## Discussion

To our knowledge, this is the first multicenter study of the trajectories of the HbA1c level and BMIz in Korean children and adolescents with T1D and T2D using OMOP CDM. Using CDM, research designs are easily shared, enabling us to conduct a multicenter study using big data while maintaining patient privacy. T1D patients showed a gradual worsening of glycemic control with an increasing trend in BMIz for 5 years after diagnosis. T2D patients also showed an increase in HbA1c levels without significant changes in BMIz during follow-up. In T1D patients, the HbA1c level and BMIz were higher in girls than in boys and in the peri-pubertal age group than in the other age groups.

We found an overall deterioration of the HbA1c level over the 5-year period after diagnosis among pediatric T1D and T2D patients. In T1D patients, the HbA1c level reached a nadir within the first 3 months and gradually increased thereafter, as in previous reports^20,21^, and a minority of patients (10.4%) achieved the recommended HbA1c target of < 7.0% by the fifth year. A recent report from the United States showed that only 17% of youth with T1D achieved an HbA1c level of < 7.5%, and 21% of adults achieved an HbA1c level of < 7.0%^22^. Despite significant advances in T1D management, including intensive insulin therapy and diabetes technologies, achieving the recommended target HbA1c level in youth with T1D remains challenging.

More than half of the T2D patients failed to achieve the glycemic target after 5 years, comparable with previous studies^23,24^. Several studies have evaluated real-world longitudinal trends in the HbA1c level in youth with T2D^23–26^, and reported worsening of glycemic control from 1 to 1.5 years after diagnosis^25,26^. In this study, the HbA1c level began to increase at 6–12 months after diagnosis, suggesting a rapid decline in β-cell function in children and adolescents with T2D^27^. Although T2D in youth has a higher risk of complications with rapid worsening compared with adults with T2D or patients with T1D^28,29^, management is hampered by the limited treatment options^12^ or poor compliance with therapy^30,31^. Considering the increasing burden of pediatric diabetes and the poor metabolic control rate, further efforts to improve glycemic control, including ongoing education and monitoring systems, are needed.

Heterogeneity in the HbA1c trajectory among youth with T1D according to sex, puberty, BMI, ethnicity, and treatment status has been reported in western countries^15–17^. T1D patients in this study also showed a different glycemic control trajectory according to sex or age group, with worse glycemic control in girls and peri-pubertal patients than in boys and younger patients, respectively, in line with previous reports^15,20,32^. Although the results were not significant due to the small number of patients, T2D patients in this study showed similar patterns. Girls have intrinsically higher insulin resistance than boys^33^, and girls with T1D have a higher risk of cardiovascular disease than boys with T1D^34^. Also, worsening of metabolic control during puberty is a major problem in pediatric diabetes care, attributable to physiological insulin resistance^33^, increased autonomy in diabetes management, and mental health issues^35^. Our finding of a prominent increase in the HbA1c level in the 5–9-year age group with T1D may be due to puberty, and the minimal increase in the HbA1c level in the 0–4-year age group may reflect the impact of parents on the diabetes care of young children. Considering the importance of glycemic control from an early age to prevent chronic microvascular complications, an improved metabolic control strategy is needed for high-risk patients such as pubertal girls.

T1D patients experienced an increase in BMIz over 5 years, characterized by an initial rapid increase for a few months after diagnosis and a gradual increase thereafter, consistent with previous reports^20,21,36–38^. Rapid weight gain immediately after T1D diagnosis is common^37,38^ and is caused by recovery of premorbid BMI by rehydration and the anabolic effect of insulin treatment. However, a long-term increase in BMI in T1D patients can lead to insulin resistance^10^, poor glycemic control^39^, dyslipidemia, hypertension^10,40^, and an increased risk of cardiovascular disease^10^. The prevalence of childhood obesity has dramatically increased worldwide^41^, and similar findings have been observed in T1D^39,40,42^. A recent multinational study reported that up to 30% of children and adolescents with T1D were overweight or obese^42^. In this study, 21% of T1D patients were overweight and/or obese 5 years after the diagnosis, similar to the frequency in the general population of Korea^43^. Because obesity has become a common problem in children and adolescents with T1D, monitoring BMI during the treatment course should be emphasized as a key component of pediatric T1D care.

In this study, peri-pubertal girls with T1D showed a higher BMIz. Girls usually have a higher total body fat content and experience more weight gain during puberty than boys^44^, and similar findings were observed in pediatric T1D patients^36,37^. An increased leptin level and hyperinsulinemia were associated with weight gain in pubertal girls, but not in boys, with T1D^45^. Also, puberty has been suggested as a critical period for weight gain in pediatric T1D^8,15^. We observed a prominent increase in BMIz among the 5–9-year age group at 3 years after diagnosis, suggesting an effect of puberty. The BMI trajectory is heterogeneous in youth with T1D, differing by sex, age at diabetes onset, and ethnicity^15,18,46^, indicating the need to identify risk groups and provide education on weight control, especially for pubertal girls.

Although T2D patients in this study showed weight loss for a few months, they failed to maintain that weight reduction. Data from the Treatment Options for type 2 diabetes in Adolescents and Youth (TODAY) study also revealed an increasing BMI trend over 5 years of follow-up, even with lifestyle interventions^25^. Sustained weight reduction is difficult in youth with T2D, attributable to poor compliance with lifestyle therapy^30^. However, weight loss in children and adolescents with T2D has shown positive effects on metabolic control^14^ and cardiovascular outcomes^13^. Considering the importance of weight control in T2D patients, further efforts to identify those at risk of an increase in BMIz and ongoing lifestyle education are needed.

Although we could not evaluate the statistical association between the HbA1c level and BMIz trajectories, the overall patterns of the two measurements were similar, suggesting a positive relationship. However, the relationship between the HbA1c level and BMI is complex in pediatric diabetic patients, and previous analyses of their association are inconsistent^9,39^. Excessive energy intake along with inadequate insulin treatment causes an increase in the HbA1c level and BMI, while suboptimal glycemic control with chronic hyperglycemia may lead to weight loss, resulting in an increase in the HbA1c level and a decrease in BMI. A recent study reported a U-shaped association between the HbA1c level and BMIz in children and adolescents with T1D^42^, indicating the importance of appropriate weight control.

This study has several limitations. First, there was a risk of inconsistency in the data collection and entry among the hospitals, depending on the frequency of patient visits and the data-input method. The mapping from source code to standard vocabularies for CDM conversion may also differ among the institutions. To exclude the risk of inconsistency among the institutions, we standardized all concepts among institutions for conversion of the source code. Second, the CDM database lacks detailed clinical information on pubertal status, insulin regimen and dosing, use of a diabetes device, and socioeconomic status, which could affect glycemic control and BMI trends. Although the treatment regimen is an important factor for metabolic control in patients with diabetes, we could not evaluate the influence of medications in this study. Various OHDSI groups are working to convert data including those related to oncology, clinical trials, psychiatry, and medical devices, into CDM format. Therefore, it is expected that more detailed data will be included in the CDM in the future. Third, a small number of patients was followed for 5 years, which prevented assessment of the changes in HbA1c level and BMIz. Although several statistical methods can overcome these limitations, such as mixed models and survival analysis, they are not applicable to CDM-based distributed research networks due to limited access to individual-level data. However, subgroup analyses among patients with complete paired data also revealed significant changes in the HbA1c level and BMIz. The database for the CDM deidentifies personal information and lacks additional clinical data such as previous medical history. Therefore, we could not identify and exclude patients referred from other hospitals. The baseline data of these patients in the database may not represent the values at the time of initial diagnosis. Further investigations are needed to overcome these limitations of CDM-based research. We attempted to minimize these limitations using large-scale data from three EHR-based standardized CDM databases.

In conclusion, this is the first large-scale multicenter study using CDM of the longitudinal trajectories of the HbA1c level and BMIz in Korean children and adolescents with diabetes. Both T1D and T2D patients showed deterioration in glycemic control during the 5-year period after diagnosis, and T1D patients experienced significant increase in BMIz during follow-up. Pubertal girls with T1D showed worse trajectories of the HbA1c level and BMIz compared with boys with T1D or younger patients. Because both optimal glycemic control and maintaining an appropriate weight during treatment are key for diabetes care, insight into the overall trends of HbA1c level and BMI can improve diabetes management. Further studies are needed to identify the trajectory patterns and predictors of glycemic control and BMI changes in pediatric T1D and T2D patients.

## Methods

### Data sources

We used observational health data transformed into CDMs from three tertiary hospitals in the Seoul metropolitan area of Korea: Seoul National University Bundang Hospital (SNUBH), Seoul National University Hospital (SNUH), and Asan Medical Center (AMC). The CDM of each hospital consists of de-identified patient-level EHR data routinely collected during medical services. In this study, we used EHR data from outpatients, inpatients, and emergency patients obtained between May 2003 and December 2018 at SNUBH, October 2004 and February 2019 at SNUH, and January 1998 and December 2017 at AMC. The study protocol was approved by the institutional review boards (SNUBH: no. X-1811-507-901; SNUH: no. 1811-182-988; AMC: no. S2018-2400-0001), and informed consent was exempted from institutional review boards. All aspects of the study were conducted in accordance with the Declaration of Helsinki.

### Study population and design

Patients aged < 15 years with at least one diagnosis of T1D or T2D and available anthropometric measurements (height and weight) or HbA1c data at diagnosis were included (Supplementary Fig. S3). The index date of diabetes diagnosis was defined as any first exposure to insulin or hypoglycemic drug. The type of diabetes was classified according to the International Classification of Diseases 10th revision (ICD-10) codes registered at the last follow-up (E10 for T1D and E11 for T2D). Patients were categorized into three groups according to age at diagnosis: 0–4, 5–9, and 10–14 years. Anthropometric measurements and HbA1c levels for 5 years after diagnosis (0, 3, 6, 12, 24, 36, 48, and 60 months) were investigated. BMI was calculated as weight divided by height in meters squared (kg/m^2^). For children aged < 2 years, weight-for-height values were calculated instead of BMI. The z-scores of BMI and weight-for-height were determined based on the 2017 Korean National Growth Charts^47^. Overweight and obesity was defined as 1.0364 ≤ BMIz < 1.6449 (85th–94th percentile of BMI for age and sex) and BMIz ≥ 1.6449 (≥ 95th percentile of BMI for age and sex), respectively. For those aged < 2 years, overweight was defined as weight-for-height z-score ≥ 1.6449 (≥ 95th percentile of weight-for-height for age and sex). We labeled patients with overweight and/or obesity as “overweight/obesity group” for further analysis. Patients in the good glycemic control group had an HbA1c < 7%, the target of the American Diabetes Association^6^ and International Society for Pediatric and Adolescents Diabetes^7^. The codes and concepts used in this study are listed in Supplementary Table S7.

### Statistical analysis

Continuous variables were tested for normality and are presented as means with standard deviation. Categorical variables are presented as frequencies with percentages. For comparison of variables between groups, Student’s *t*-test or analysis of variance was used for continuous variables and the chi-squared test was used for categorical variables. A paired *t*-test was performed to compare changes in the HbA1c level or BMIz at 3 months (baseline) with those at 36 or 60 months in patients with available paired data. Because these values were compared between groups at each time point, we applied Bonferroni correction for multiple testing to minimize error. We merged the data of continuous variables from the three hospitals to calculate the mean values of HbA1c and BMIz. Data visualization was conducted to identify the trajectories of HbA1c and BMIz in pediatric diabetic patients. Statistical analyses were performed using the R statistical software package (version 3.6.0; R Foundation for Statistical Computing, Vienna, Austria)^48^, and the tidyverse R package^49^ was used to preprocess and ggplot2 R package^50^ was used to visualize the data. Values of *p* < 0.05 were considered indicative of statistical significance.

## Supplementary Information

Supplementary Information 1.

Supplementary Information 2.

Supplementary Information 3.

Supplementary Information 4.

## Data Availability

The datasets generated during the current study are not publicaly available due to the unique characteristics of common data model analysis but available from the corresponding author on reasonable request.
