# Supplementary material for: Trajectories in glycated hemoglobin and body mass index in children and adolescents with diabetes using the common data model
Source: Sci Rep. 2021 Jul 16;11:14614. doi: 10.1038/s41598-021-94194-5 (PMC8285411; doi:10.1038/s41598-021-94194-5)
Supplement: Supplementary file 1 — Supplementary Information 1. [file 41598_2021_94194_MOESM1_ESM.docx]

**Trajectories in Glycated Hemoglobin and Body Mass Index in Children and Adolescents with Diabetes Using the Common Data Model**

Yun Jeong Lee^1,2^, Sooyoung Yoo^3^, Soyoung Yi^3^, Seok Kim^3^, Chunggak Lee^3^, Jihoon Cho^3^, Soyeon Ahn^4^, Sunkyu Choi^4,5^, Hee Hwang^2,3,13^, Young Ah Lee^1,2^, Choong Ho Shin^1,2^, Hyung-Jin Yoon^6^, Kwangsoo Kim^7,8^, Eunhye Song^8^, Jin Ho Choi^9^, Han Wook Yoo^9^, Young-Hak Kim^10,11,12^, Ji Seon Oh^10^, Eun-Ae Kang^12^, Ga Kyoung Baek^12^, Jae Hyun Kim^2,13^ **^*^**

**Affiliations:**

^1^Department of Pediatrics, Seoul National University Children’s Hospital, Seoul, Korea; ^2^Department of Pediatrics, Seoul National University College of Medicine, Seoul, Korea; ^3^Office of eHealth Research and Businesses, Seoul National University Bundang Hospital, Seongnam, Gyeonggi-do, Korea; ^4^Division of Statistics, Medical Research Collaborating Center, Seoul National University Bundang Hospital, Seongnam, Gyeonggi-do, Korea; ^5^Department of Biostatistics, Korea University College of Medicine, Seoul, Korea; ^6^Department of Biomedical Engineering, Seoul National University College of Medicine, Seoul, Korea; ^7^Transdisciplinary Department of Medicine and Advanced Technology, Seoul National University Hospital, Seoul, Korea; ^8^Department of Data Science Research, Innovative Medical Technology Research Institute, Seoul National University Hospital, Seoul, Korea; ^9^Department of Pediatrics, Asan Medical Center, University of Ulsan College of Medicine, Seoul, Korea; ^10^Department of Information Medicine, Asan Medical Center, University of Ulsan College of Medicine, Seoul, Korea; ^11^Division of Cardiology, Department of Internal Medicine, Asan Medical Center, University of Ulsan College of Medicine, Seoul, Korea; ^12^Health Innovation Big Data Center, Asan Institute of Life Sciences, Asan Medical Center, Seoul, Korea; ^13^Department of Pediatrics, Seoul National University Bundang Hospital, Seongnam, Gyeonggi-do, Korea

**^*^Corresponding author:**

Jae Hyun Kim, M.D., PhD;

Department of Pediatrics, Seoul National University Bundang Hospital, Seongnam, Gyeonggi-do, Korea;

Department of Pediatrics, Seoul National University College of Medicine, Seoul, Korea;

Tel: +82-31-787-7287;

Fax: +82-31-787-4054;

E-mail: pedendo@snubh.org

Supplementary Table S1. Medications used in patients with type 2 diabetes at each time point

|  | 3 mo | 6 mo | 12 mo | 24 mo | 36 mo | 48 mo | 60 mo |
| --- | --- | --- | --- | --- | --- | --- | --- |
| Metformin only | 133 (72.7) | 129 (77.2) | 115 (75.7) | 69 (63.3) | 48 (58.5) | 37 (53.6) | 34 (59.6) |
| Metformin with insulin | 36 (19.7) | 29 (17.4) | 27 (17.8) | 24 (22.0) | 24 (29.3) | 23 (33.3) | 17 (29.8) |
| Insulin only | 14 (7.7) | 9 (5.4) | 10 (6.6) | 16 (14.7) | 10 (12.2) | 9 (13.0) | 6 (10.5) |
| Total | 183 | 167 | 152 | 109 | 82 | 69 | 57 |

Data are presented as number (%).

Supplementary Table S2. Comparison of baseline characteristics between patients followed-up and those who were not at each time point

|  | At 3 months | | At 6 months | | At 12 months | | At 24 months | |
| --- | --- | --- | --- | --- | --- | --- | --- | --- |
|  | Follow-up | Lost to follow-up | Follow-up | Lost to follow-up | Follow-up | Lost to follow-up | Follow-up | Lost to follow-up |
| No. of patients (%) | 834 (93.8) | 55 (6.2) | 801 (90.1) | 88 (9.9) | 751 (84.5) | 138 (15.5) | 667 (75.0) | 222 (25.0) |
| Institute |  |  |  |  |  |  |  |  |
| SNUBH, n (%) | 84 (85.7)^*^ | 14 (14.3)^*^ | 78 (79.6)^*^ | 20 (20.4)^*^ | 71 (72.4)^*^ | 27 (27.6)^*^ | 57 (58.2)^*^ | 41 (41.8)^*^ |
| SNUH, n (%) | 411 (95.4)^*^ | 20 (4.6)^*^ | 396 (91.9)^*^ | 35 (8.1)^*^ | 371 (86.1)^*^ | 60 (13.9)^*^ | 332 (77.0)^*^ | 99 (23.0)^*^ |
| AMC, n (%) | 339 (94.2)^*^ | 21 (5.8)^*^ | 327 (90.8)^*^ | 33 (9.2)^*^ | 309 (85.8)^*^ | 51 (14.2)^*^ | 278 (77.2)^*^ | 82 (22.8)^*^ |
| Male, n (%) | 373 (44.7) | 20 (36.4) | 362 (45.2) | 31 (35.2) | 337 (44.9) | 56 (40.6) | 302 (45.3) | 91 (41.0) |
| Age at diagnosis, years | 10.5 (3.5) | 10.0 (3.6) | 10.5 (3.5) | 10.1 (3.6) | 10.5 (3.4) | 10.2 (3.7) | 10.5 (3.4) | 10.3 (3.6) |
| Age group |  |  |  |  |  |  |  |  |
| 0–4 years, n (%) | 92 (11.0) | 8 (14.5) | 88 (11.0) | 12 (13.6) | 81 (10.8) | 19 (13.8) | 72 (10.8) | 28 (12.6) |
| 5–9 years, n (%) | 196 (23.5) | 14 (25.5) | 187 (23.3) | 23 (26.1) | 175 (23.3) | 35 (25.4) | 151 (22.6) | 59 (26.6) |
| 10–14 years, n (%) | 546 (65.5) | 33 (60.0) | 526 (65.7) | 53 (60.2) | 495 (65.9) | 84 (60.9) | 444 (66.6) | 135 (60.8) |
| Body mass index or weight-for height z-score^†^ | 0.1 (1.5) | -0.1 (1.6) | 0.1 (1.5) | 0.1 (1.6) | 0.1 (1.6) | 0.2 (1.6) | 0.1 (1.5) | 0.3 (1.6) |
| Normal/Overweight  /Obese ^†^, n (%) | 517/57/92 (77.6/8.6/13.8) | 39/3/8 (78.0/6.0/16.0) | 495/55/88 (77.6/8.6/13.8) | 61/5/12 (78.2/6.4/15.4) | 463/47/81 (78.3/8.0/13.7) | 93/13/19 (74.4/10.4/15.2) | 413/41/66 (79.4/7.9/12.7) | 143/19/34 (73.0/9.7/17.3) |
| Glycated hemoglobin^†^, % | 9.8 (2.6) | 10.2 (2.5) | 9.8 (2.6) | 9.9 (2.6) | 9.8 (2.6) | 10.1 (2.7) | 9.9 (2.7) | 9.7 (2.5) |

|  | At 36 months | | At 48 months | | At 60 months | |
| --- | --- | --- | --- | --- | --- | --- |
|  | Follow-up | Lost to follow-up | Follow-up | Lost to follow-up | Follow-up | Lost to follow-up |
| No. of patients (%) | 580 (65.2) | 209 (34.8) | 501 (56.4) | 388 (43.6) | 451 (50.7) | 438 (49.3) |
| Institute |  |  |  |  |  |  |
| SNUBH, n (%) | 43 (43.9)^*^ | 55 (56.1)^*^ | 38 (38.8)^*^ | 60 (61.2)^*^ | 33 (33.7)^*^ | 65 (66.3)^*^ |
| SNUH, n (%) | 288 (66.8)^*^ | 143 (33.2)^*^ | 246 (57.1)^*^ | 185 (42.9)^*^ | 219 (50.8)^*^ | 212 (49.2)^*^ |
| AMC, n (%) | 249 (69.2)^*^ | 111 (30.8)^*^ | 217 (60.3)^*^ | 143 (39.7)^*^ | 199 (55.3)^*^ | 161 (44.7)^*^ |
| Male, n (%) | 263 (45.3) | 130 (42.1) | 226 (45.1) | 167 (43.0) | 202 (44.8) | 191 (43.6) |
| Age at diagnosis, years | 10.7 (3.4) | 10.1 (3.6) | 10.7 (3.4)^*^ | 10.1 (3.6)^*^ | 10.8 (3.3)^*^ | 10.2 (3.6)^*^ |
| Age group |  |  |  |  |  |  |
| 0–4 years, n (%) | 61 (10.5)^*^ | 39 (12.6)^*^ | 49 (9.8)^*^ | 51 (13.1)^*^ | 43 (9.5)^*^ | 57 (13.0)^*^ |
| 5–9 years, n (%) | 122 (21.0)^*^ | 88 (28.5)^*^ | 104 (20.8)^*^ | 106 (27.3)^*^ | 93 (20.6)^*^ | 117 (26.7)^*^ |
| 10–14 years, n (%) | 397 (68.4)^*^ | 182 (58.9)^*^ | 348 (69.5)^*^ | 231 (59.5)^*^ | 315 (69.8)^*^ | 264 (60.3)^*^ |
| Body mass index or weight-for height z-score^†^ | 0.0 (1.5) | 0.2 (1.5) | 0.0 (1.4)^*^ | 0.3 (1.6)^*^ | 0.0 (1.5) | 0.2 (1.6) |
| Normal/Overweight/Obese ^†^, n (%) | 348/33/56 (79.6/7.6/12.8) | 205/27/44 (74.3/9.8/15.9) | 301/26/40 (82.0/7.1/10.9)^*^ | 255/34/60 (73.1/9.7/17.2)^*^ | 259/24/37 (80.9/7.5/11.6) | 297/36/63 (75.0/9.1/15.9) |
| Glycated hemoglobin^†^, % | 9.9 (2.6) | 9.8 (2.7) | 9.8 (2.5) | 9.9 (2.7) | 9.8 (2.6) | 9.9 (2.6) |

Data are presented as means (standard deviation) for continuous variables and numbers (%) for categorical variables. *p*-values were derived by Student’s *t*-test (continuous variables) or the chi-squared test (categorical variables).

^†^ Body mass index z-scores and overweight prevalences were available in 713 patients, and the glycated hemoglobin level at baseline was available in 831 patients.

Asterisk (^*^) indicates *p* < 0.05 between values of patients followed-up and those who were not at each time point.

Supplementary Table S3. Mean glycated hemoglobin levels during follow-up of study participants according to diabetes type, sex, and age group.

| Category | HbA1c (%) |  | 0 mo | 3 mo | 6 mo | 12 mo | 24 mo | 36 mo | 48 mo | 60 mo |
| --- | --- | --- | --- | --- | --- | --- | --- | --- | --- | --- |
| Diabetes type | T1D | Mean | 9.99 | 7.61 | 7.82 | 8.09 | 8.32 | 8.47 | 8.63 | 8.72 |
|  |  | SD | 2.58 | 1.66 | 1.66 | 1.67 | 1.66 | 1.69 | 1.66 | 1.84 |
|  |  | N | 609 | 459 | 463 | 494 | 438 | 415 | 366 | 327 |
|  | T2D | Mean | 9.45 | 6.52 | 6.60 | 7.06 | 7.89 | 8.35 | 8.19 | 8.81 |
|  |  | SD | 2.65 | 1.16 | 1.47 | 1.67 | 2.30 | 2.37 | 2.37 | 2.47 |
|  |  | N | 222 | 159 | 144 | 150 | 122 | 100 | 79 | 74 |
|  |  | *p* | 0.008 | <0.001 | <0.001 | <0.001 | 0.428 | 0.999 | 0.975 | 0.999 |
| Sex (T1D) | T1D boys | Mean | 9.95 | 7.65 | 7.67 | 7.88 | 8.04 | 8.19 | 8.25 | 8.27 |
|  |  | SD | 2.53 | 1.70 | 1.60 | 1.59 | 1.57 | 1.59 | 1.40 | 1.60 |
|  |  | N | 260 | 202 | 199 | 205 | 187 | 182 | 164 | 142 |
|  | T1D girls | Mean | 10.03 | 7.58 | 7.93 | 8.24 | 8.53 | 8.69 | 8.94 | 9.06 |
|  |  | SD | 2.63 | 1.63 | 1.69 | 1.71 | 1.71 | 1.73 | 1.79 | 1.94 |
|  |  | N | 349 | 257 | 264 | 289 | 251 | 233 | 202 | 185 |
|  | *p* |  | 0.999 | 0.999 | 0.703 | 0.133 | 0.015 | 0.019 | <0.001 | 0.001 |
| Age group (T1D) | 0–4 years | Mean | 9.49 | 7.60 | 7.63 | 7.77 | 7.72 | 7.78 | 7.85 | 7.83 |
|  |  | SD | 2.02 | 1.29 | 1.09 | 1.25 | 1.07 | 0.97 | 1.22 | 1.13 |
|  |  | N | 88 | 64 | 67 | 77 | 69 | 62 | 56 | 47 |
|  | 5–9 years | Mean | 9.79 | 7.24 | 7.45 | 7.75 | 8.05 | 8.27 | 8.55 | 8.77 |
|  |  | SD | 2.69 | 1.23 | 1.17 | 1.19 | 1.26 | 1.29 | 1.30 | 1.68 |
|  |  | N | 163 | 139 | 133 | 151 | 128 | 106 | 89 | 78 |
|  | 10–14 years | Mean | 10.21 | 7.81 | 8.05 | 8.37 | 8.64 | 8.73 | 8.86 | 8.91 |
|  |  | SD | 2.64 | 1.90 | 1.93 | 1.94 | 1.91 | 1.91 | 1.82 | 1.97 |
|  |  | N | 358 | 256 | 263 | 266 | 241 | 247 | 221 | 202 |
|  | *p* |  | 0.265 | 0.039 | 0.013 | 0.002 | <0.001 | 0.001 | 0.002 | 0.010 |
| Sex (T2D) | T2D boys | Mean | 9.63 | 6.35 | 6.44 | 7.02 | 7.54 | 8.49 | 8.11 | 8.42 |
|  |  | SD | 2.60 | 1.03 | 1.32 | 1.54 | 1.95 | 2.55 | 2.64 | 2.46 |
|  |  | N | 115 | 85 | 78 | 78 | 58 | 47 | 41 | 38 |
|  | T2D girls | Mean | 9.25 | 6.71 | 6.79 | 7.11 | 8.21 | 8.24 | 8.29 | 9.22 |
|  |  | SD | 2.70 | 1.27 | 1.62 | 1.80 | 2.55 | 2.21 | 2.07 | 2.45 |
|  |  | N | 107 | 74 | 66 | 72 | 64 | 53 | 38 | 36 |
|  | *p* |  | 0.999 | 0.449 | 0.999 | 0.999 | 0.839 | 0.999 | 0.999 | 0.999 |
| Age group (T2D) | 5–9 years | Mean | 8.58 | 6.93 | 6.57 | 7.23 | 8.02 | 8.46 | 7.41 | 8.04 |
|  |  | SD | 2.61 | 1.62 | 1.16 | 1.55 | 2.86 | 2.99 | 1.49 | 2.05 |
|  |  | N | 29 | 22 | 20 | 21 | 19 | 12 | 9 | 7 |
|  | 10–14 years | Mean | 9.58 | 6.45 | 6.61 | 7.04 | 7.86 | 8.34 | 8.29 | 8.89 |
|  |  | SD | 2.63 | 1.06 | 1.52 | 1.69 | 2.20 | 2.29 | 2.45 | 2.51 |
|  |  | N | 193 | 137 | 124 | 129 | 103 | 88 | 70 | 67 |
|  | *p* |  | 0.505 | 0.999 | 0.999 | 0.999 | 0.999 | 0.999 | 0.999 | 0.999 |

HbA1c, glycated hemoglobin; T1D, type 1 diabetes; T2D, type 2 diabetes

Bonferroni-corrected *p*-values were calculated to compare values at each time point between groups.

Supplementary Table S4. Paired comparisons of glycated hemoglobin levels at 3 versus 36 or 60 months.

| HbA1c (%) | Category  (n) | 3 mo | 36 mo | *p* | No. | 3 mo | 60 mo | *p* |
| --- | --- | --- | --- | --- | --- | --- | --- | --- |
| T1D | Total  (305) | 7.62 (1.68) | 8.49 (1.73) | < 0.001 | Total  (226) | 7.78 (1.71) | 8.69 (1.81) | < 0.001 |
| Sex | Boys  (140) | 7.61 (1.65) | 8.20 (1.57) | 0.002 | Boys  (102) | 7.71 (1.70) | 8.29 (1.57) | 0.013 |
|  | Girls  (165) | 7.63 (1.72) | 8.72 (1.83) | < 0.001 | Girls  (124) | 7.84 (1.73) | 9.02 (1.93) | < 0.001 |
| Age group | 0–4 years  (40) | 7.65 (1.46) | 7.84 (1.05) | 0.505 | 0–4 years  (27) | 7.47 (1.45) | 7.70 (1.27) | 0.553 |
|  | 5–9 years  (83) | 7.23 (1.30) | 8.27 (1.28) | < 0.001 | 5–9 years  (59) | 7.30 (1.44) | 8.75 (1.56) | < 0.001 |
|  | 10–14 years  (182) | 7.80 (1.85) | 8.73 (1.97) | < 0.001 | 10–14 years  (140) | 8.05 (1.82) | 8.85 (1.94) | < 0.001 |
| T2D | Total  (76) | 6.42 (1.18) | 8.27 (2.40) | < 0.001 | Total  (50) | 6.43 (0.92) | 8.46 (2.46) | < 0.001 |
| Sex | Boys  (38) | 6.27 (0.93) | 8.31 (2.40) | < 0.001 | Boys  (28) | 6.44 (0.95) | 8.32 (2.42) | < 0.001 |
|  | Girls  (38) | 6.58 (1.38) | 8.23 (2.43) | < 0.001 | Girls  (22) | 6.42 (0.89) | 8.65 (2.55) | < 0.001 |
| Age group | 5–9 years  (9) | 6.76 (2.19) | 9.02 (3.26) | 0.105 | 5–9 years  (5) | 6.66 (1.15) | 7.79 (2.18) | 0.345 |
|  | 10–14 years  (67) | 6.38 (0.99) | 8.16 (2.27) | < 0.001 | 10–14 years  (45) | 6.41 (0.90) | 8.54 (2.50) | < 0.001 |

HbA1c, glycated hemoglobin; T1D, type 1 diabetes; T2D, type 2 diabetes

Values are means (standard deviation). *p-*values were derived by paired *t*-test (within-group comparisons), and Bonferroni-corrected *p*-values were used for multiple comparisons.

Supplementary Table S5. Mean body mass index z-scores during follow-up of participants according to diabetes type, sex, and age group.

| Category | BMIz |  | 0 mo | 3 mo | 6 mo | 12 mo | 24 mo | 36 mo | 48 mo | 60 mo |
| --- | --- | --- | --- | --- | --- | --- | --- | --- | --- | --- |
| Diabetes type | T1D | Mean | -0.37 | -0.08 | -0.14 | -0.01 | 0.01 | 0.05 | 0.25 | 0.27 |
|  |  | SD | 1.18 | 0.96 | 1.01 | 1.03 | 1.00 | 0.99 | 0.95 | 1.08 |
|  |  | N | 539 | 325 | 315 | 369 | 317 | 272 | 247 | 199 |
|  | T2D | Mean | 1.53 | 1.45 | 1.25 | 1.58 | 1.54 | 1.29 | 1.51 | 1.39 |
|  |  | SD | 1.43 | 1.50 | 1.52 | 1.64 | 1.58 | 1.52 | 1.58 | 1.43 |
|  |  | N | 174 | 95 | 98 | 90 | 79 | 64 | 48 | 51 |
|  | *p* |  | <0.001 | <0.001 | <0.001 | <0.001 | <0.001 | <0.001 | <0.001 | <0.001 |
| Sex (T1D) | T1D boys | Mean | -0.41 | -0.15 | -0.26 | -0.17 | -0.11 | -0.09 | 0.14 | 0.05 |
|  |  | SD | 1.24 | 0.98 | 1.06 | 1.02 | 1.07 | 1.01 | 0.96 | 1.05 |
|  |  | N | 229 | 148 | 139 | 152 | 135 | 123 | 113 | 94 |
|  | T1D girls | Mean | -0.33 | -0.02 | -0.04 | 0.10 | 0.11 | 0.17 | 0.34 | 0.47 |
|  |  | SD | 1.14 | 0.93 | 0.96 | 1.03 | 0.94 | 0.96 | 0.94 | 1.08 |
|  |  | N | 310 | 177 | 176 | 217 | 182 | 149 | 134 | 105 |
|  | *p* |  | 0.999 | 0.999 | 0.506 | 0.094 | 0.428 | 0.267 | 0.753 | 0.042 |
| Age group (T1D) | 0–4 years | Mean | 0.22 | 0.57 | 0.15 | 0.59 | 0.16 | 0.08 | 0.08 | -0.18 |
|  |  | SD | 1.75 | 1.19 | 1.37 | 1.31 | 1.21 | 1.15 | 0.87 | 0.88 |
|  |  | N | 82 | 47 | 46 | 56 | 45 | 42 | 32 | 30 |
|  | 5–9 years | Mean | -0.45 | -0.09 | -0.13 | -0.11 | -0.05 | -0.13 | 0.20 | 0.25 |
|  |  | SD | 1.04 | 0.86 | 0.86 | 0.85 | 0.91 | 0.82 | 0.90 | 1.01 |
|  |  | N | 157 | 102 | 99 | 111 | 94 | 77 | 65 | 46 |
|  | 10–14 years | Mean | -0.48 | -0.24 | -0.22 | -0.12 | 0.01 | 0.14 | 0.31 | 0.39 |
|  |  | SD | 1.00 | 0.87 | 0.97 | 0.99 | 0.99 | 1.01 | 0.99 | 1.13 |
|  |  | N | 300 | 176 | 170 | 202 | 178 | 153 | 150 | 123 |
|  | *p* |  | <0.001 | <0.001 | 0.682 | <0.001 | 0.999 | 0.999 | 0.999 | 0.268 |
| Sex (T2D) | T2D boys | Mean | 1.37 | 1.29 | 1.21 | 1.40 | 1.43 | 1.43 | 1.68 | 1.23 |
|  |  | SD | 1.38 | 1.46 | 1.52 | 1.69 | 1.61 | 1.60 | 1.66 | 1.33 |
|  |  | N | 90 | 57 | 55 | 47 | 38 | 34 | 24 | 28 |
|  | T2D girls | Mean | 1.69 | 1.69 | 1.30 | 1.77 | 1.65 | 1.13 | 1.34 | 1.57 |
|  |  | SD | 1.48 | 1.55 | 1.53 | 1.58 | 1.56 | 1.43 | 1.51 | 1.56 |
|  |  | N | 84 | 38 | 43 | 43 | 41 | 30 | 24 | 23 |
|  | *p* |  | 0.999 | 0.999 | 0.999 | 0.999 | 0.999 | 0.999 | 0.999 | 0.999 |
| Age group (T2D) | 5–9 years | Mean | 1.47 | 1.56 | 0.72 | 1.44 | 1.49 | 1.50 | 0.60 | 1.16 |
|  |  | SD | 2.09 | 2.29 | 2.06 | 2.15 | 2.12 | 2.30 | 2.24 | 2.69 |
|  |  | N | 21 | 16 | 17 | 15 | 14 | 8 | 9 | 5 |
|  | 10–14 years | Mean | 1.53 | 1.43 | 1.36 | 1.61 | 1.55 | 1.26 | 1.72 | 1.41 |
|  |  | SD | 1.33 | 1.31 | 1.37 | 1.54 | 1.46 | 1.40 | 1.34 | 1.28 |
|  |  | N | 153 | 79 | 81 | 75 | 65 | 56 | 39 | 46 |
|  | *p* |  | 0.999 | 0.999 | 0.999 | 0.999 | 0.999 | 0.999 | 0.999 | 0.999 |

BMIz, body mass index z-score; T1D, type 1 diabetes; T2D, type 2 diabetes

Bonferroni-corrected *p*-values were calculated to compare values at each time point between groups.

Supplementary Table S6. Paired comparisons of body mass index z-scores at 3 versus 36 or 60 months.

| BMIz | Category (n) | 3 mo | 36 mo | *p* | No. | 3 mo | 60mo | *p* |
| --- | --- | --- | --- | --- | --- | --- | --- | --- |
| T1D | Total  (183) | −0.13 (0.96) | 0.03 (0.94) | 0.097 | Total  (126) | −0.11 (1.00) | 0.25 (1.05) | 0.005 |
| Sex | Boys  (86) | −0.19 (0.97) | −0.02 (0.98) | 0.256 | Boys  (61) | −0.26 (0.89) | 0.06 (1.04) | 0.064 |
|  | Girls  (97) | −0.08 (0.95) | 0.09 (0.91) | 0.227 | Girls  (65) | 0.03 (1.08) | 0.43 (1.03) | 0.032 |
| Age group | 0–4 years  (20) | 0.86 (1.40) | −0.21 (1.30) | 0.017 | 0–4 years  (17) | 0.88 (1.31) | −0.06 (1.00) | 0.043 |
|  | 5–9 years  (53) | −0.17 (0.76) | −0.11 (0.78) | 0.681 | 5–9 years  (31) | −0.07 (0.85) | 0.15 (0.98) | 0.347 |
|  | 10–14 years  (110) | −0.29 (0.85) | 0.15 (0.93) | < 0.001 | 10–14 years  (81) | −0.30 (0.90) | 0.34 (1.08) | < 0.001 |
| T2D | Total  (37) | 1.26 (1.38) | 1.22 (1.70) | 0.905 | Total  (26) | 1.30 (1.23) | 1.18 (1.38) | 0.748 |
| Sex | Boys  (21) | 1.33 (1.45) | 1.33 (1.83) | 0.997 | Boys  (15) | 1.40 (1.03) | 1.26 (1.32) | 0.735 |
|  | Girls  (16) | 1.17 (1.32) | 1.07 (1.55) | 0.849 | Girls  (11) | 1.15 (1.50) | 1.07 (1.51) | 0.908 |
| Age group | 5–9 years  (5) | 0.95 (2.28) | 0.93 (2.43) | 0.989 | 5–9 years  (3) | 0.28 (2.86) | 0.15 (2.81) | 0.958 |
|  | 10–14 years  (32) | 1.31 (1.23) | 1.27 (1.60) | 0.897 | 10–14 years  (23) | 1.43 (0.91) | 1.31 (1.13) | 0.704 |

BMIz, body mass index z-scores; T1D, type 1 diabetes; T2D, type 2 diabetes

Values are means (standard deviation). *p-*values were derived by paired *t*-test (within-group comparisons), and Bonferroni-corrected *p*‑values were calculated for multiple comparisons.

Supplementary Table S7. List of concept sets

| OMOP  concept Id | Concept name | Domain | Vocabulary | Excluded | Descendants |
| --- | --- | --- | --- | --- | --- |
| *Type of diabetes concept sets* | | | | | |
| 201254 | Type 1 diabetes mellitus | Condition | SNOMED | NO | YES |
| 201826 | Type 2 diabetes mellitus | Condition | SNOMED | NO | YES |
| *Insulin or hypoglycemic drug concept sets* | | | | | |
| 21600713 | Insulins and analogues | Drug | ATC | NO | YES |
| 21600744 | Blood glucose lowering drugs, excl. insulins | Drug | ATC | NO | YES |
| *Body height concept set* | | | | | |
| 3036277 | Body height | Measurement | LOINC | NO | NO |
| *Body weight concept set* | | | | | |
| 3013762 | Body weight measured | Measurement | LOINC | NO | NO |
| *Body mass index concept set* | | | | | |
| 3038553 | Body mass index (BMI) [ratio] | Measurement | LOINC | NO | NO |
| *Glycated hemoglobin concept set* | | | | | |
| 3005673 | Hemoglobin A1c/hemoglobin. total in blood by HPLC | Measurement | LOINC | NO | NO |

**Supplementary figure legends**

Supplementary Figure S1. Changes in the proportion of the good glycemic control group (HbA1c < 7%) among patients with type 1 diabetes and type 2 diabetes from diagnosis to 60 months after diagnosis.

Supplementary Figure S2. Changes in the proportion of the overweight/obesity group among patients with type 1 diabetes and type 2 diabetes from diagnosis to 60 months after diagnosis.

Supplementary Figure S3. Flowchart of the study population
