# Supplementary figures and images for: Trajectories in glycated hemoglobin and body mass index in children and adolescents with diabetes using the common data model
Source: Sci Rep. 2021 Jul 16;11:14614. doi: 10.1038/s41598-021-94194-5 (PMC8285411; doi:10.1038/s41598-021-94194-5)

# Supplementary Figure S1

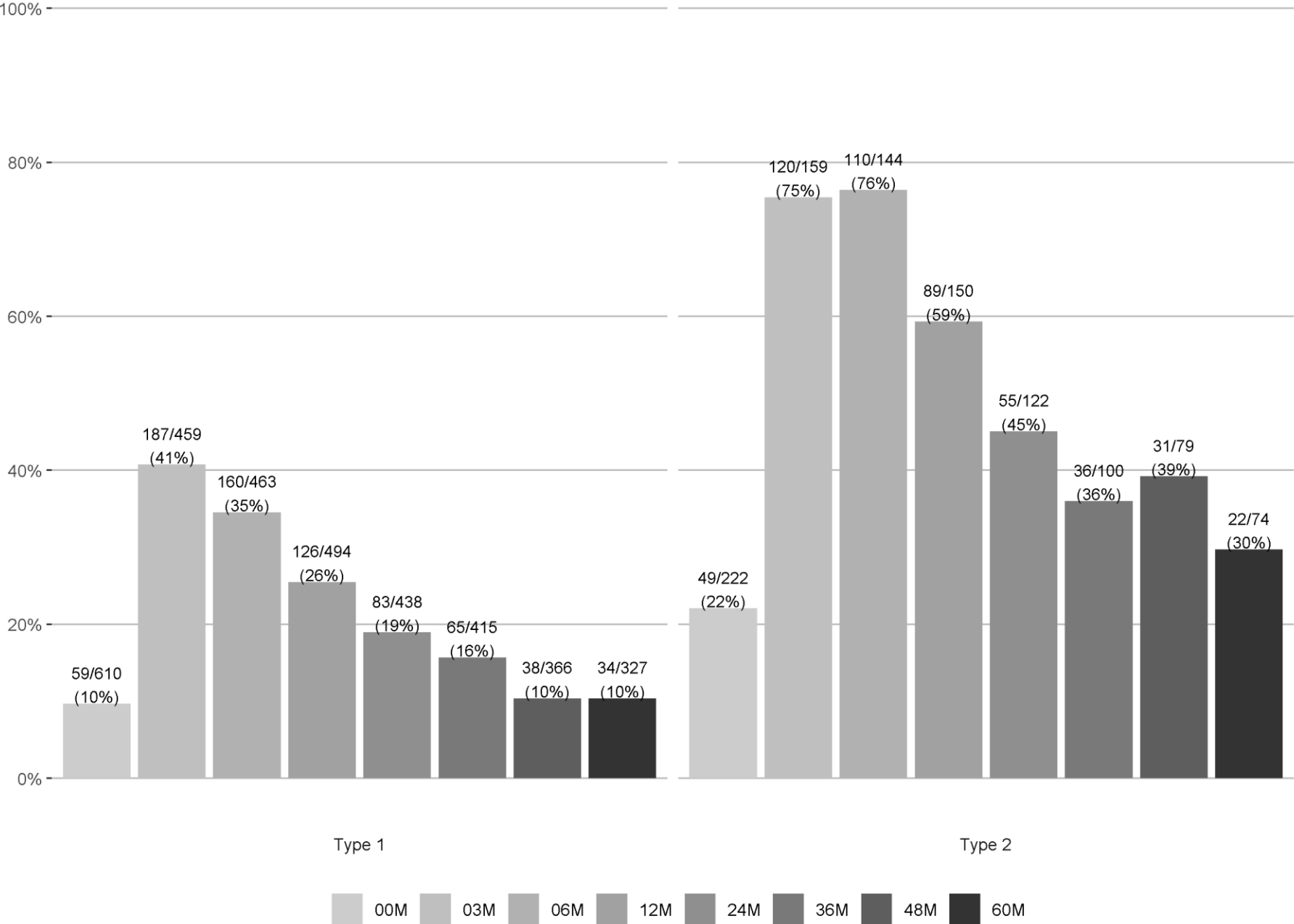

Supplement: Supplementary file 2 — Supplementary Information 2. [file 41598_2021_94194_MOESM2_ESM.pdf]

# Supplementary Figure S2

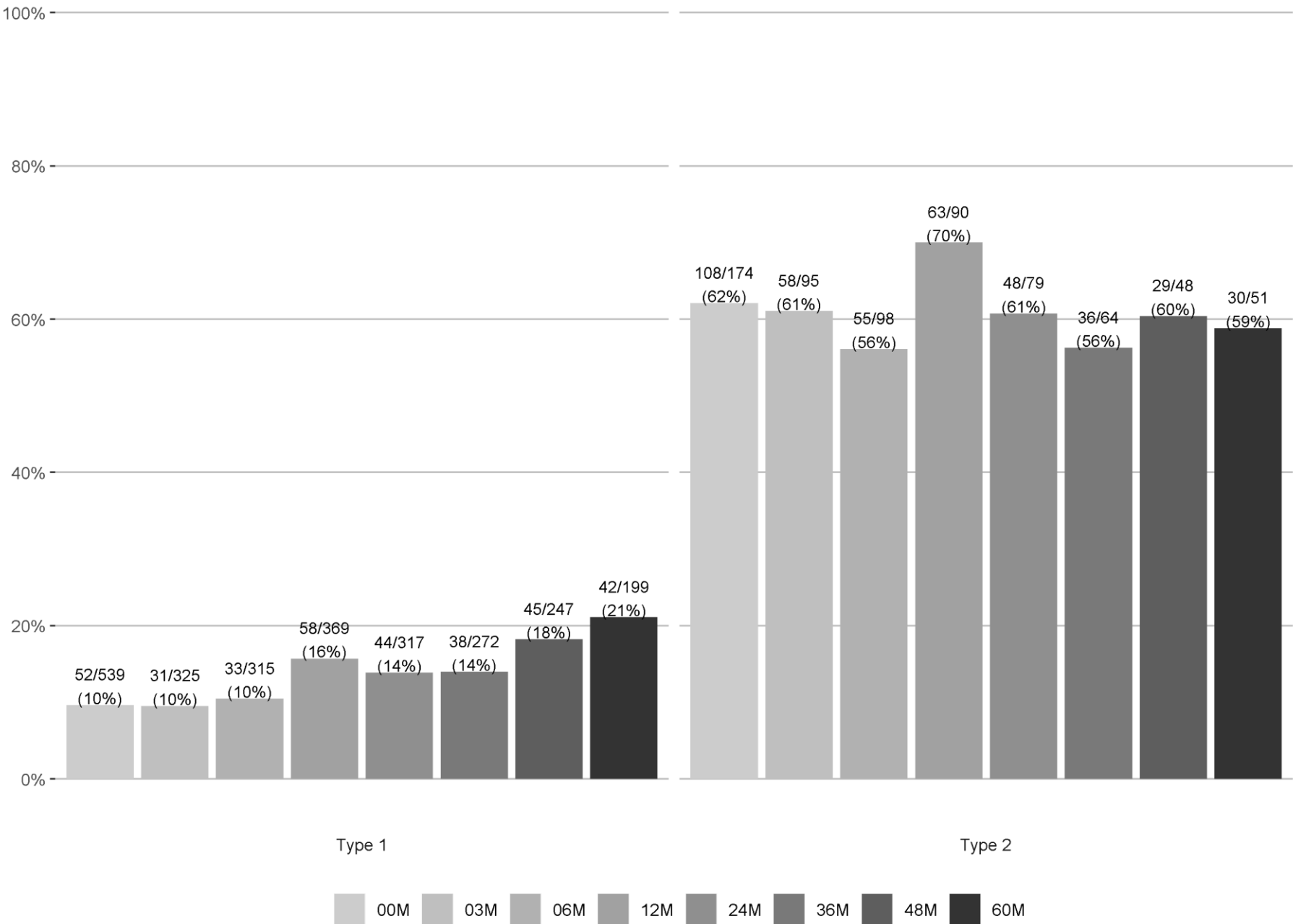

Supplement: Supplementary file 3 — Supplementary Information 3. [file 41598_2021_94194_MOESM3_ESM.pdf]

# Supplementary Figure S3

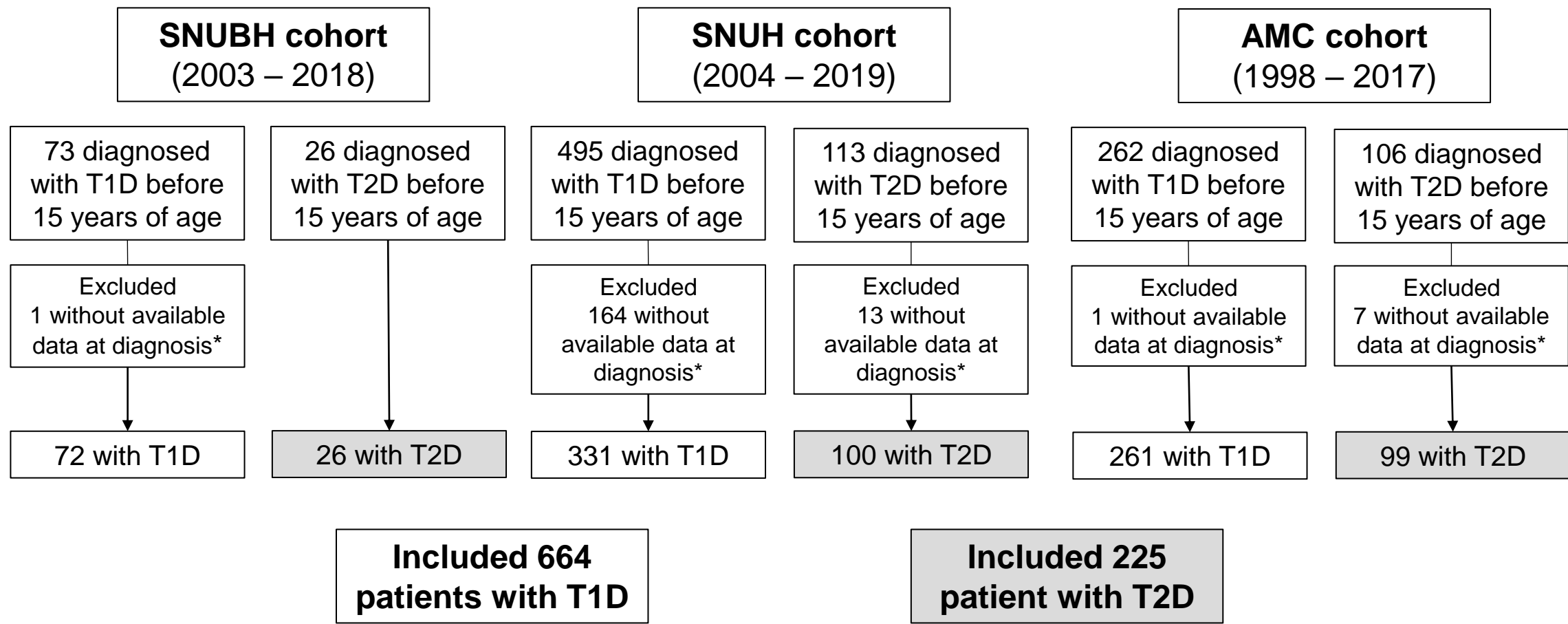

Supplement: Supplementary file 4 — Supplementary Information 4. [file 41598_2021_94194_MOESM4_ESM.pdf]
